# Supplementary material for: Nanometric flow and earthquake instability
Source: Nat Commun. 2021 Nov 22;12:6779. doi: 10.1038/s41467-021-26996-0 (PMC8608838; doi:10.1038/s41467-021-26996-0)
Supplement: Supplementary file 2 — Supplementary Information [file 41467_2021_26996_MOESM2_ESM.pdf]

Supplementary Information for

Nanometric flow and earthquake instability

Hongyu Sun[1*] and Matej Pec[1*]

[1] Department of Earth, Atmospheric and Planetary Sciences, Massachusetts Institute of Technology, 77 Massachusetts Ave, Cambridge, MA 02139, USA

[*] Corresponding Authors: hongyus@mit.edu (H. Sun) or mpec@mit.edu (M. Pec)

This Supplementary Information file includes:

Supplementary Figs. 1 to 9

Supplementary Table 1

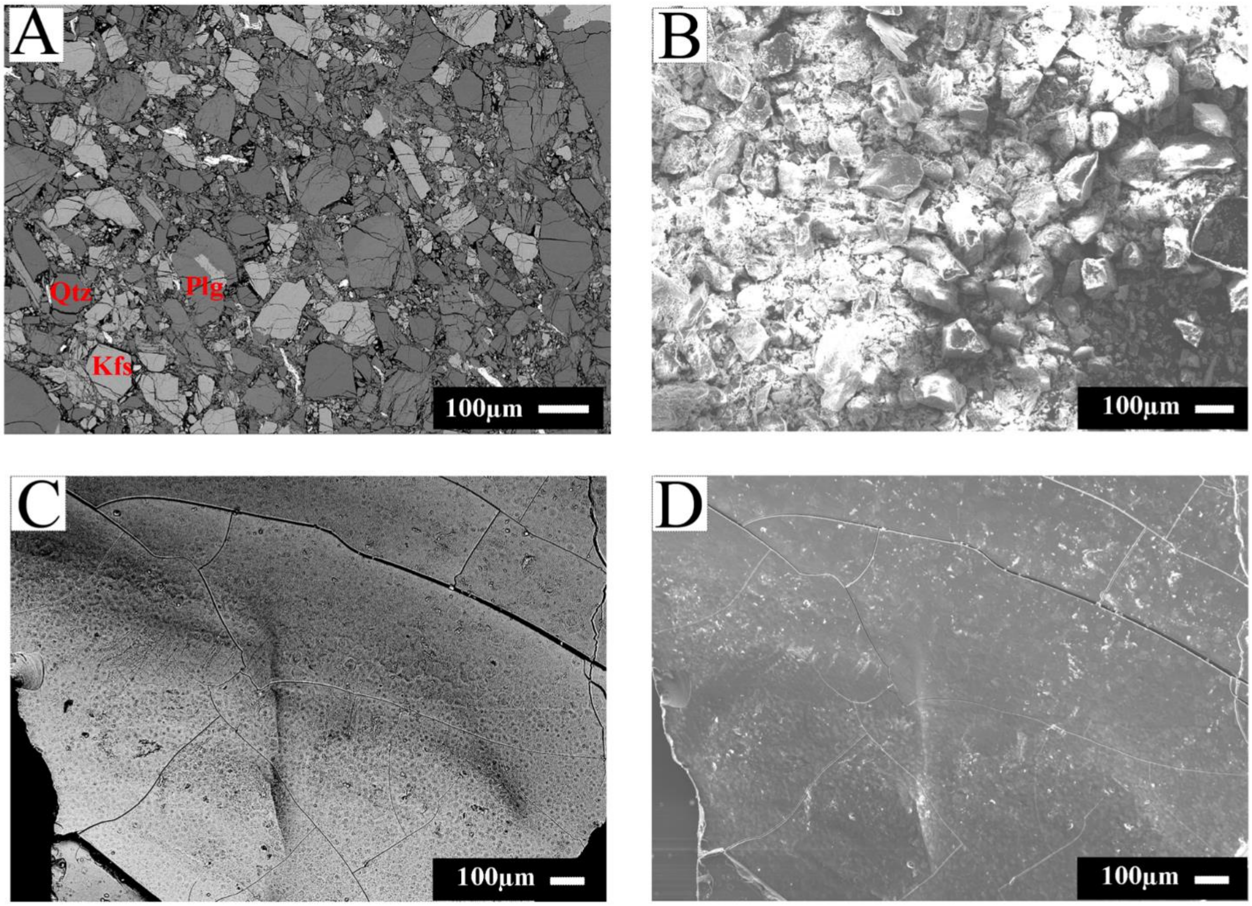

Supplementary Fig. 1 Microstructural comparison of coarse-grained Verzasca gneiss powder and ball milled Verzasca gneiss nanomaterial. a SEM-BSE image and **b** SE topographic image of the coarse-grained powder. **c** SEM-BSE image and **d** SE image of the ball milled nanomaterial.

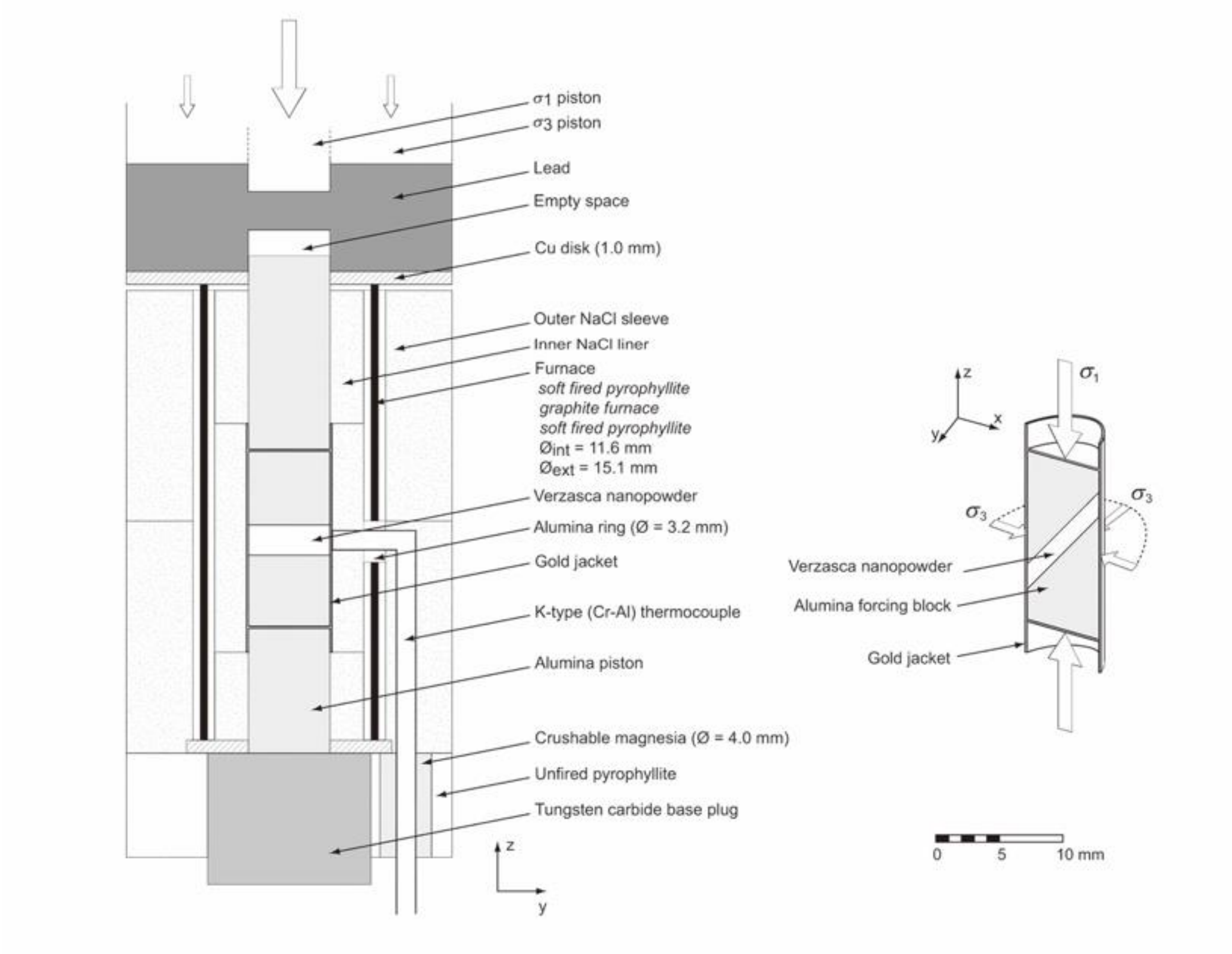

Supplementary Fig. 2 Sample assembly inside the pressure vessel and sample geometry modified after ref. 12.

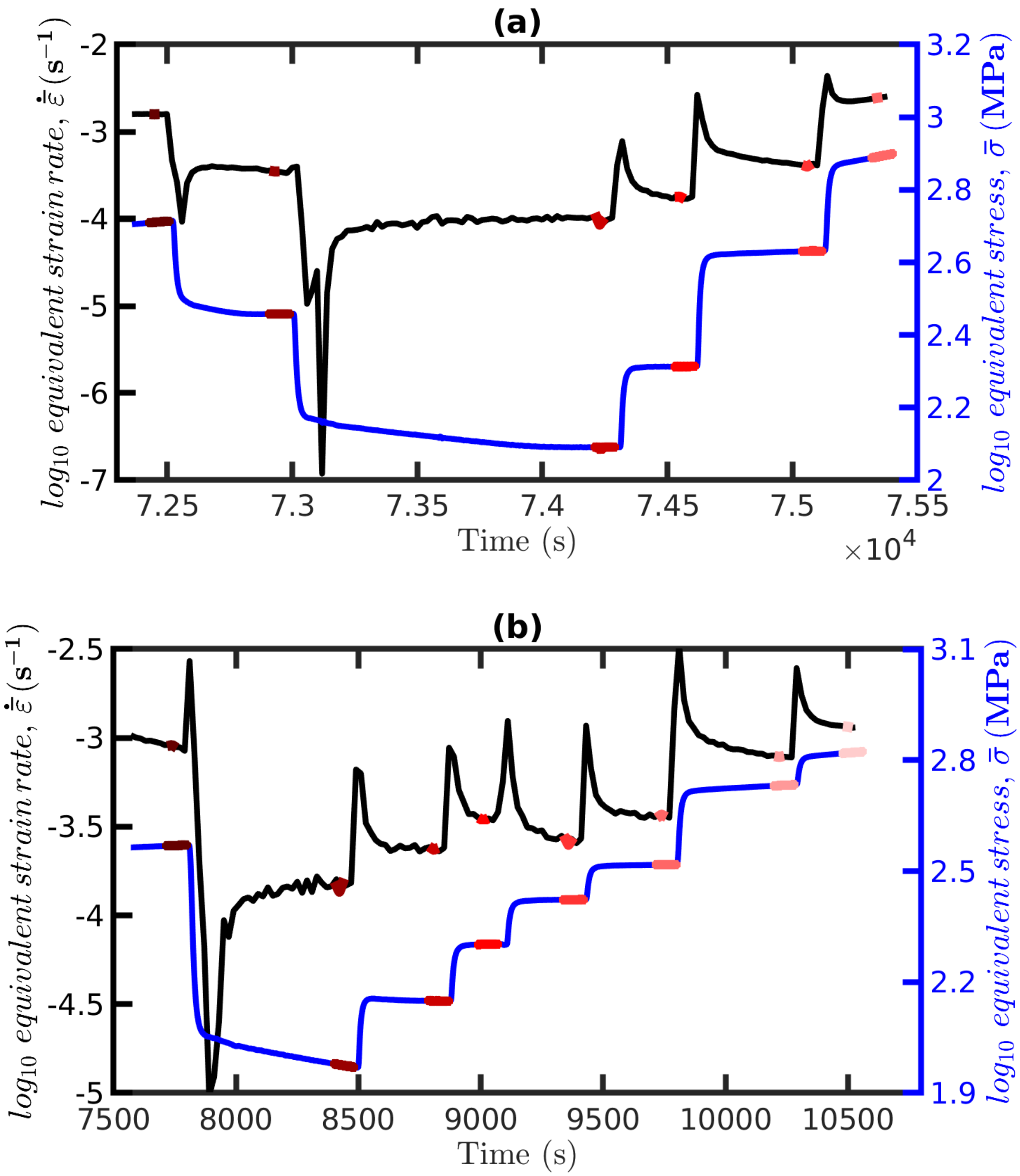

Supplementary Fig. 3 Mechanical data from two load-stepping experiments. a Experiment 042HS (500℃). **b** Experiment 064HS (300℃). The strain rate is calculated by first down-sampling the strain over a 20-s time window and then calculating the slope of strain vs. time curves. Reddish lines show the 80-s time intervals selected at the end of each step to calculate $\dot{\bar{\varepsilon}}$ and $\bar{\sigma}$ under each constant load. In total, experiment 042HS has six steps; experiment 064HS has eight. Table 1 reports the mean values of the mechanical data from these steps.

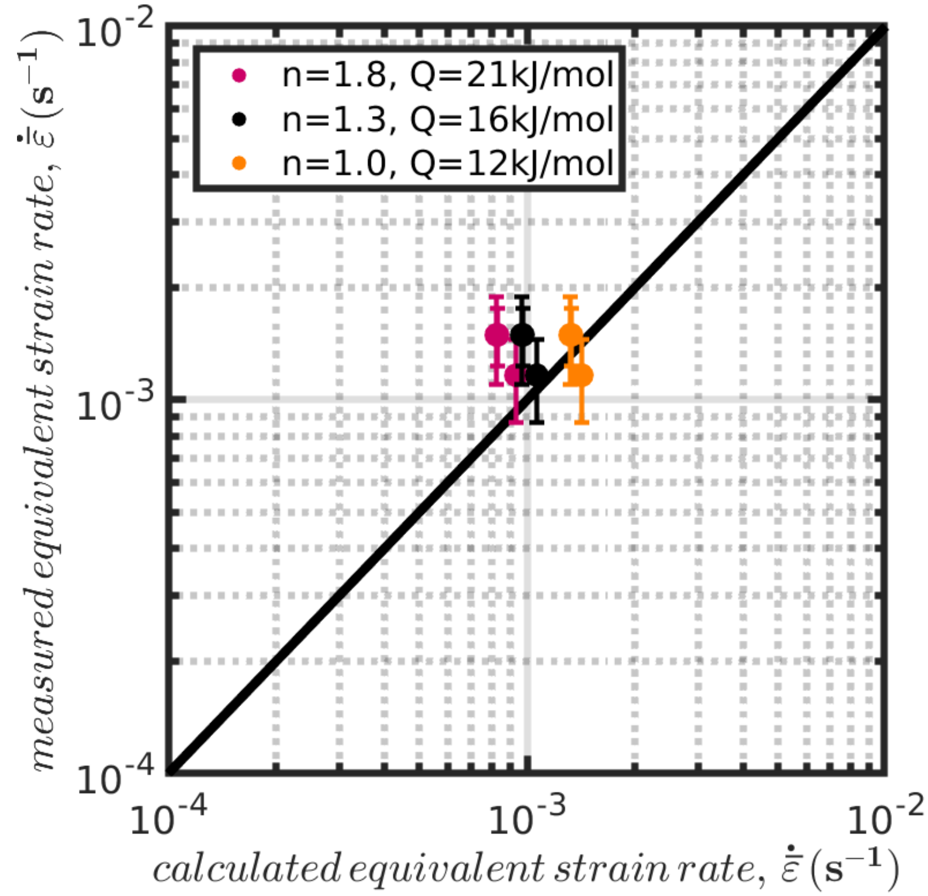

Supplementary Fig. 4 Validation of the rheological parameters. Equivalent strain rates measured in the constant-displacement-rate experiments (028HS, 030HS and 034HS) are compared with those predicted by the experimental flow law. The error bars represent the standard deviation of measurements. Supplementary Table 1 reports the rheological parameters and calculated equivalent strain rates.

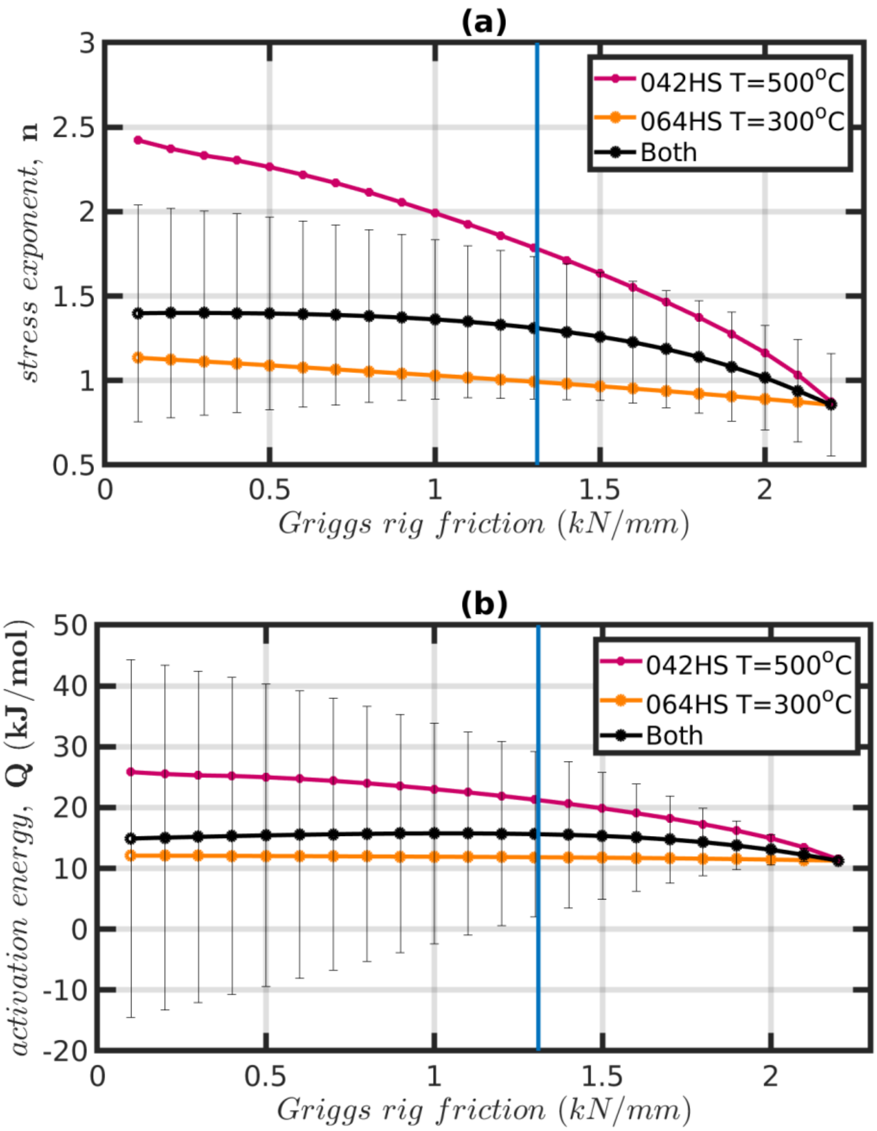

Supplementary Fig. 5 Evaluation of rheology parameters with different friction correction coefficients. a Stress exponent. **b** Activation energy. The friction correction coefficient is assumed to range from 0.1-2.2 kN/mm. (A coefficient larger than 2.3 kN/mm leads to a negative equivalent stress in the load-stepping experiment.) Error bars indicate a 95% confidence interval of the estimated rheological parameters using measurements from both experiments). Note that the negative Q in the error bars shows relatively large errors in this evaluation. The blue line indicates the evaluation reported in the main text using a friction correction coefficient of 1.31 kN/mm.

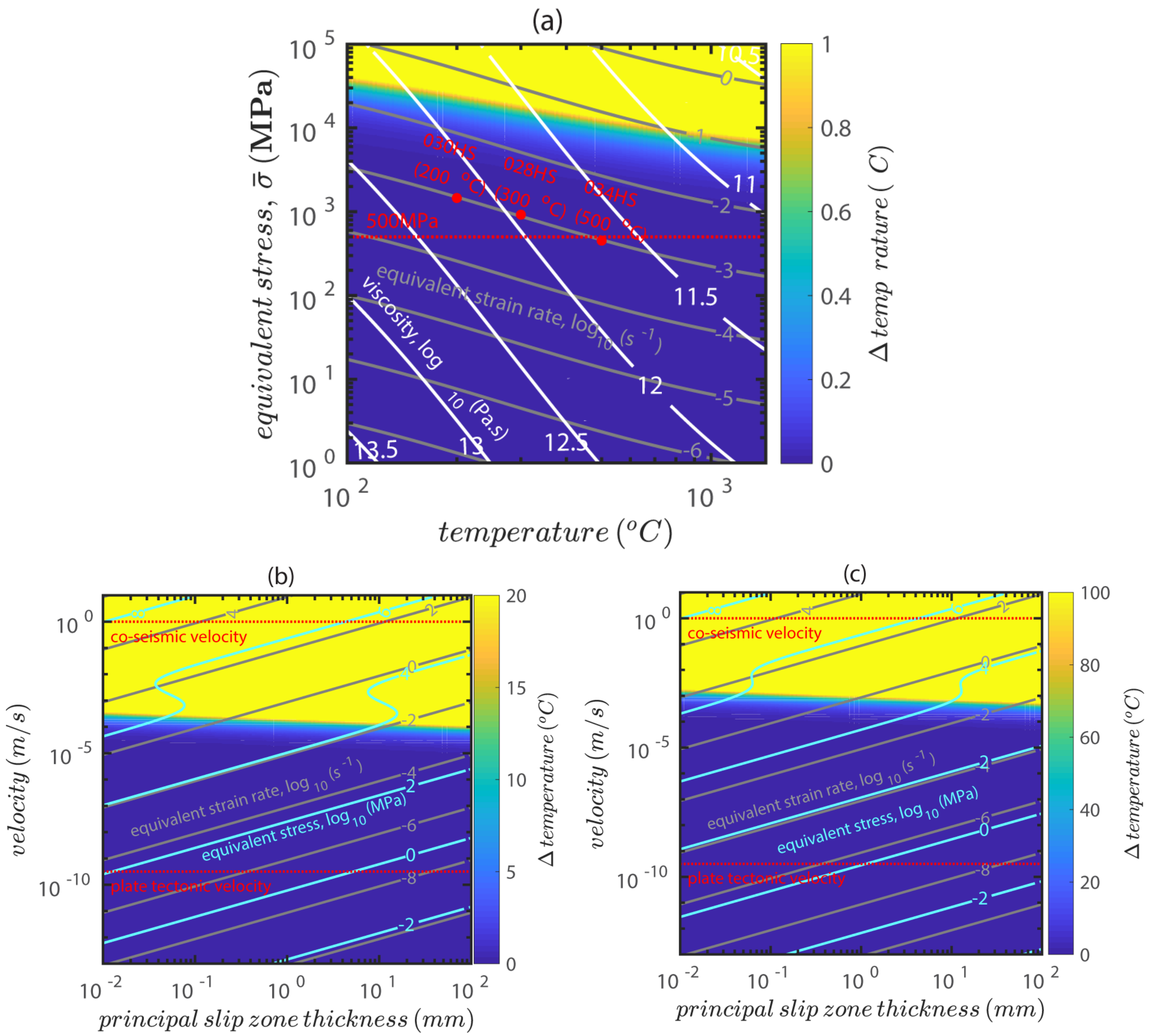

Supplementary Fig. 6 Shear heating calculations. a Shear heating calculated for the experimental samples. The experiments on nanometric fault rocks can be treated as isothermal. **b** Shear heating at an ambient temperature of 200˚C. **c** Shear heating at an ambient temperature of 500˚C. Note that the effect of shear heating becomes more pronounced with decreasing ambient temperature.

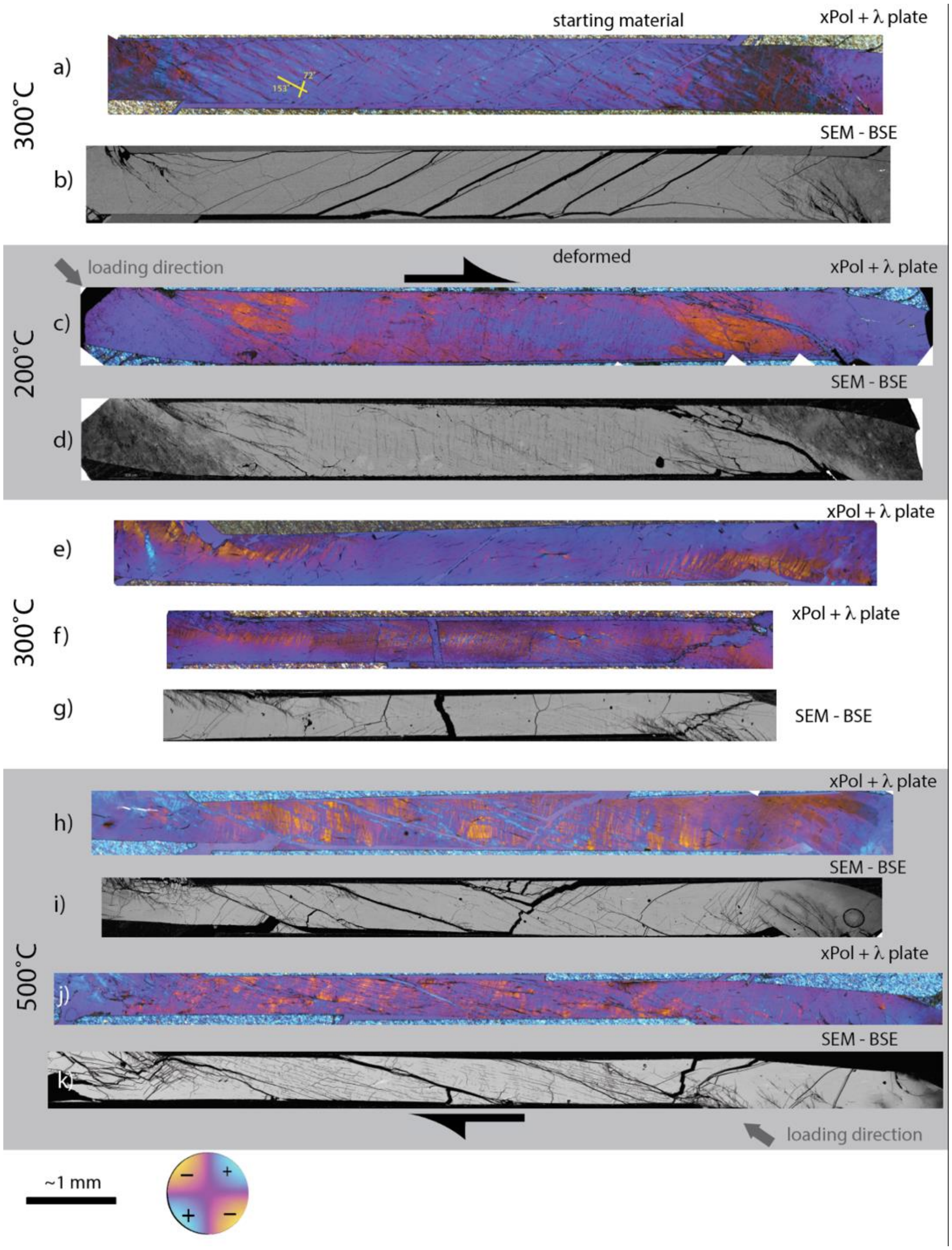

Supplementary Fig. 7 Microstructures of the shear zones in SEM-BSE and cross-polarized light with a λ plate inserted. **a** Sample pressurized and heated to run conditions, but not deformed. Notice the prominent kink bands. **b** Uniform gray suggests homogenous composition / density. **c** and **d** Sample deformed at 200˚C, notice the prominent R_1 and R_2 shears. Wider range of z-contrast in BSE images suggests a more chemically heterogenous and/or dense material than at higher T. **e** Constant load stepping experiment. At low strains, the kink bands and R_1 shears are localized to the outer ends of the shear zone boundary. **f** and **g** Higher-strain sample, R_1 shears and kinks in R_2 direction propagate through the whole lengths of the shear zone. **h** and **i** Constant displacement rate experiment. Notice the prominent kinks ("tiger-stripe patterns"). **j** and **k** Stress-stepping experiment to high strain. The pattern from lower strain experiment is repeated at higher spatial frequency.

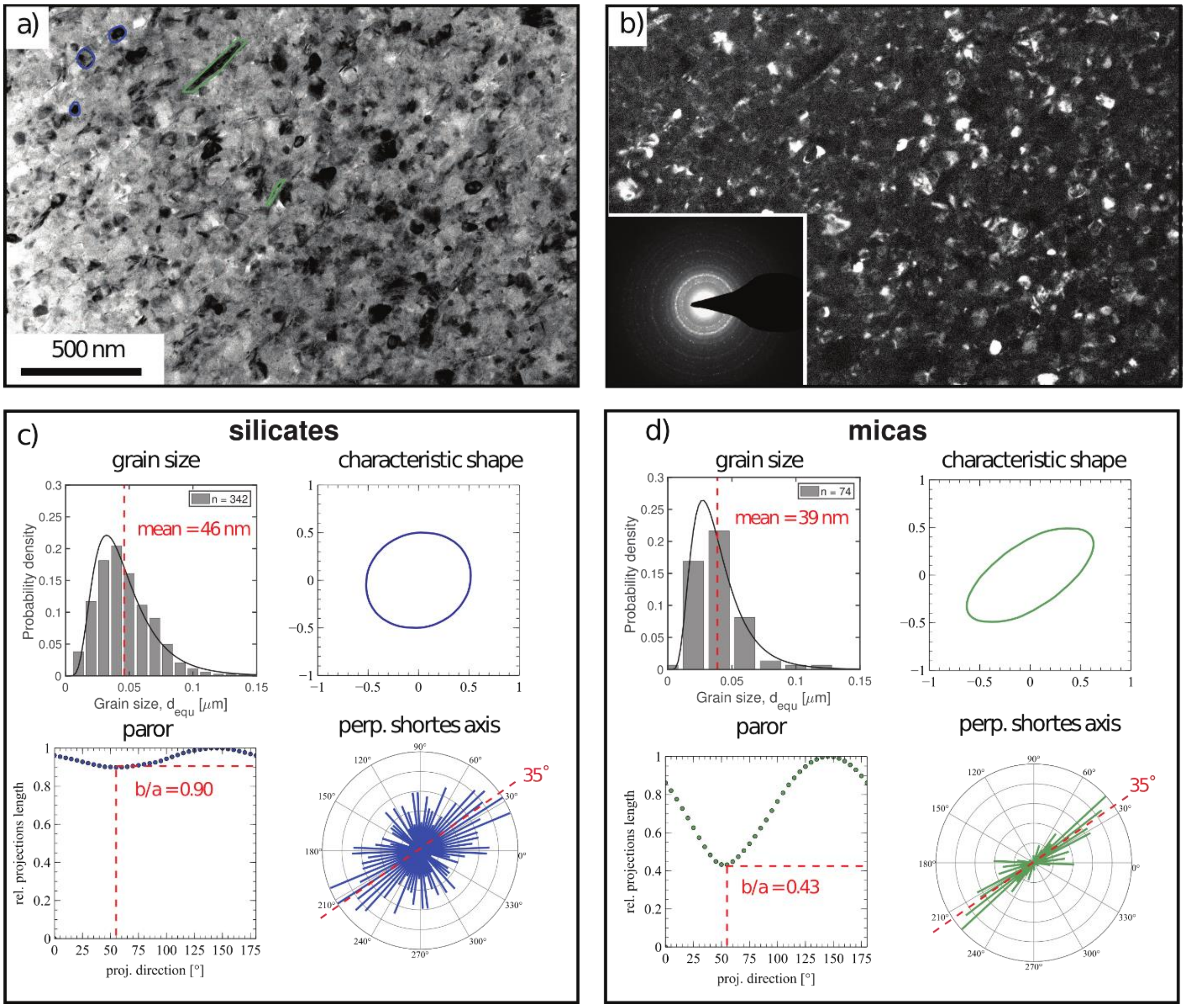

Supplementary Fig. 8 Nanostructures after deformation at 500˚C (034HS). TEM foils were cut out of thin sections using a focused ion beam (FIB). The sense of shear in the image is in and out of the screen. **a** TEM – bright field image. Notice the dense nature of the material, fine-grain size, roundish silicate grains (feldspars and quartz, highlighted in blue) and elongated mica grains (biotite and white mica, highlighted in green). **b** TEM – dark field image of same area as a). Inset shows selected area diffraction pattern documenting the nanocrystalline nature of the material. **c** Quantitative image analysis of silicate grains. Top row: grain size distribution histogram and characteristic shape of grains as determined by the surfor method (supplementary ref. 1), bottom row: strength of shape preferred orientation (SPO) and rose diagram of SPO as determined by the paror method (supplementary ref. 1). Note that silicate grains are extremely fine grained at mean d_{equ} =46 nm, rounded with a weak SPO (b/a = 0.9 where b/a = 1 is a circle). **d** Quantitative image analysis of mica grains. Top row: grain size distribution histogram and characteristic shape of grains as determined by the surfor method (supplementary ref. 1), bottom row: strength of SPO and rose diagram of SPO as determined by the paror method (supplementary ref. 1). Note that mica grains are extremely fine grained at mean d_{equ} =39 nm, similar to the silicate grains. They display a characteristic elliptical shape and strong SPO (b/a = 0.43). The orientation of the micas is consistent with the orientation of silicate grains at 35˚.

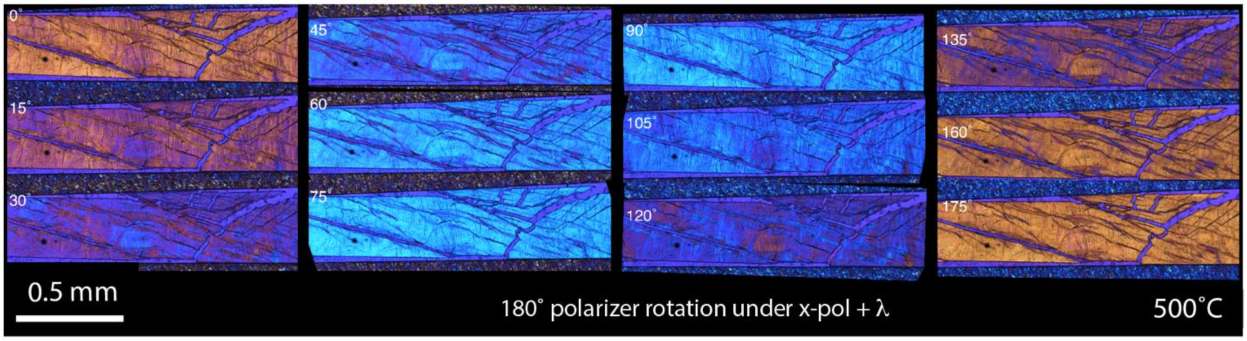

Supplementary Fig. 9 Optical anisotropy under polarizer rotation over 180˚. Experiment 034HS. Note the large areas over which optical anisotropy is coherent.

Supplementary Table 1 Validation of the rheological parameters [a]

Experiment	$\boldsymbol{n}$	$\boldsymbol{Q}$ $(\boldsymbol{kJ/mol})$	$\boldsymbol{A}$ $(\boldsymbol{MPa \cdot s^{-1}})$	calculated $\dot{\bar{\boldsymbol{\varepsilon}}}$ $(\boldsymbol{s^{-1}})$		
				**200℃** **030HS**	**300℃** **028HS**	**500℃** **034HS**
**064HS [b]**	1.0	12	1.95×10^{-5}	1.32×10^{-3}	1.42×10^{-3}	1.33×10^{-3}
**042HS [b]**	1.8	21	4.24×10^{-7}	8.19×10^{-4}	9.30×10^{-4}	8.26×10^{-4}
**Both [c]**	1.3	16	3.69×10^{-6}	9.67×10^{-4}	1.06×10^{-3}	9.73×10^{-4}

a: The maximum equivalent stress is used to calculate $\dot{\bar{\varepsilon}}$ at different temperatures.
b: A is estimated from the intercept of the $log_{10}\dot{\bar{\varepsilon}}\ vs.\ log_{10}\bar{\sigma}$ curve.
c: The temperature-independent A is estimated from the intercept of the $log_{10}\bar{\sigma}\ vs.\ 1/T$ curve with a constant $\dot{\bar{\varepsilon}}$ of 10^{-3} s^{-1}.

Supplementary Reference

1. Heilbronner, R. & Barrett, S. *Image analysis in Earth Sciences: microstructures and textures of earth materials* **129**. Springer (2013).
